# Antibacterial Potential of Nanocrystalline Zinc–Cobalt Ferrite

**DOI:** 10.3390/nano15171318

**Published:** 2025-08-28

**Authors:** Riya Panja, Tapas Kumar Bhattacharyya, Aditya Paul, Saibal Ray, Ahmed Abd El Wahed, Arianna Ceruti, Siddhartha Narayan Joardar

**Affiliations:** 1 Government College of Engineering and Ceramic Technology, P.O., Beleghata, Kolkata 700010, West Bengal, India; panja.riya2018@gmail.com (R.P.); tkb_ceramics@yahoo.co.in (T.K.B.); 2 Department of Veterinary Microbiology, West Bengal University of Animal and Fishery Sciences, P.O., Belgachia, Kolkata 700037, West Bengal, India; adityapaul28@gmail.com; 3 Centre for Cosmology, Astrophysics and Space Science (CCASS), GLA University, Mathura 281406, Utter Pradesh, India; saibal.ray@gla.ac.in; 4 Institute of Animal Hygiene and Veterinary Public Health, Leipzig University, An den Tierkliniken 1, D-04103 Leipzig, Germany; ahmed.abd_el_wahed@uni-leipzig.de

**Keywords:** antimicrobial resistance, bacteria, disk diffusion assay, minimum inhibitory concentration, nanoparticles

## Abstract

Purpose: The synthesis of nanoscale particles with antibacterial properties has garnered significant attention in pharmaceutical research, driven by the escalating threat of antibiotic-resistant bacteria. This study investigates the antibacterial efficacy of Zn–Co ferrite nanoparticles against virulent, antibiotic-resistant, and biofilm-forming strains of *Escherichia coli.* Methods: Three nanoparticle variants—S1 (Zn_0.7_Co_0.3_Fe_2_O_4_), S2 (Zn_0.5_Co_0.5_Fe_2_O_4_), and S3 (Zn_0.3_Co_0.7_Fe_2_O_4_)—were synthesized using the solution combustion method by systematically varying the Zn:Co molar ratio. The Scanning Electron Micrograph, X-ray diffraction analysis, Complementary Fourier-transform infrared, Minimum Inhibitory Concentration, and Minimum Bactericidal Concentration were performed. Results: The SEM spectroscopy study revealed distinct morphological differences as a function of the cobalt substitution level within the spinel ferrite matrix. At the highest level of cobalt substitution (Zn_0.3_Co_0.7_Fe_2_O_4_), the microstructure displayed significant irregularities, with enhanced agglomeration and a notably broader particle size distribution. X-ray diffraction analysis confirmed the formation of crystalline structures, with an average crystallite size of 12.65 nm. Complementary Fourier-transform infrared spectroscopy revealed characteristic absorption bands in the 400–600 cm^−1^ range, indicative of the cubic spinel structure of the ferrite nanoparticles. The higher-frequency band was associated with metal–oxide stretching in the tetrahedral sites, while the lower-frequency band corresponded to stretching in the octahedral sites. The Minimum Inhibitory Concentration and Minimum Bactericidal Concentration assays revealed that Zn–Co ferrite nanoparticles possess potent antibacterial activity against virulent, antibiotic-resistant, and biofilm-forming strains of *E. coli*. Conclusion: Increasing the molar ratio of Zn to Co enhances the antibacterial activity of the nanoparticles. These findings suggest that Zn–Co ferrite nanoparticles could serve as a promising alternative to conventional antibacterial agents for combating multidrug-resistant pathogenic bacteria in the future.

## 1. Introduction

The global rise of Antimicrobial Resistance (AMR) among pathogenic bacteria poses a significant threat to both human and animal health, with profound implications for the treatment of infectious diseases. This issue is increasingly well-documented and continues to escalate worldwide. Bacteria, which are essential components of the ecosystem, are ubiquitous and primarily free-living organisms [1]. However, many bacterial species are pathogenic, capable of causing a wide range of infectious diseases. These include conditions such as cholera, pneumonia, enteritis, colitis, cystitis, meningitis, syphilis, anthrax, leprosy, tuberculosis, tetanus, bubonic plague, as well as more common infections like boils and acne.

Antibiotics have played a pivotal role in modern medicine, effectively treating bacterial infections and saving countless lives. Common classes such as *penicillins*, *cephalosporins*, *tetracyclines*, and *fluoroquinolones* have been widely used to combat pathogenic bacteria. However, the World Health Organization (WHO) has recently highlighted a critical global health concern: the alarming rise in antibiotic resistance among common bacterial strains (Antimicrobial Resistance: Global Report on Surveillance, WHO). This resistance poses a significant threat, as it has been documented in both pathogenic and non-pathogenic microorganisms, including *Salmonella* spp., *Campylobacter* spp., *Yersinia* sp., *Escherichia coli* O157:H7, *enterococci*, *Pasteurella* sp., and *Actinobacillus* sp., with varying degrees of severity [2].

The first documented case of antibacterial resistance dates back to 1930, emerging in response to sulfonamides [3]. Notably, resistance to β-lactam antibiotics was observed even prior to the clinical introduction of penicillin, with the first β-lactamase enzyme identified in *Escherichia coli* [4].

The primary mechanism of bacterial resistance to β-lactam antibiotics involves the production of β-lactamases, enzymes that hydrolyze and inactivate these drugs [5]. Notable examples include extended-spectrum β-lactamases (ESBLs), plasmid-mediated AmpC β-lactamases, and carbapenemases, which confer resistance to broad-spectrum β-lactams. Additionally, biofilm formation serves as a critical virulence factor, further enhancing antibiotic tolerance and resistance development. Among the over 300 known β-lactamases, the most clinically significant variants are predominantly expressed by Gram-negative bacteria [6].

To address the growing challenge of antibiotic resistance, researchers are actively exploring alternatives to conventional antibiotics. Among these, nanomaterials have emerged as a promising candidate due to their unique ability to circumvent resistance mechanisms. Defined as materials with structural components in the size range of 1–100 nm, nanomaterials exhibit distinct physical, chemical, and biological properties, including enhanced material strength, altered solubility, modified conductivity, tunable optical characteristics, and improved catalytic activity compared to their bulk counterparts [7]. These properties arise from their high surface-to-volume ratio and the increased proportion of surface atoms, which dominate their reactivity and functionality. Such unique attributes position nanomaterials as a viable and innovative strategy to combat antibiotic-resistant pathogens [8].

With the progress of nanotechnology and molecular biology, nanoparticles have been widely studied and applied in biomedicine and magnetic nanoparticles have become one of the research hotspots in the nanomedical field [9]. Extensive research has demonstrated the antibacterial potential of nanomaterials against pathogenic bacteria. Notably, cobalt ferrite nanoparticles (CoFe_2_O_4_) have been shown to significantly enhance the antimicrobial efficacy of chlorhexidine when used against *Enterococcus faecium*. This synergistic effect is attributed to the nanoparticles’ high surface-to-volume ratio, which facilitates greater interaction with bacterial cells [10]. Several clinically relevant pathogens, including *Escherichia coli*, *Staphylococcus aureus*, *Mycobacterium tuberculosis*, and *Pseudomonas aeruginosa*, demonstrated greater susceptibility to zinc ferrite (ZnFe_2_O_4_) nanoparticles compared to conventional antibiotics. This enhanced antimicrobial activity is attributed to two key factors: (1) the generation of reactive oxygen species (ROS) and (2) the reduced ionic size of ZnFe_2_O_4_ nanoparticles, which facilitates improved bacterial membrane penetration [11]. In a comparative study, ZnO nanoparticles showed excellent bactericidal potential against both Gram-positive (*Staphylococcus aureus* and *Bacillus subtilis*) and Gram-negative (*Escherichia coli* and *Pseudomonas aeruginosa*) bacteria while Fe_2_O_3_ nanoparticles exhibited the least bactericidal activity [12]. Biosynthesized silver nanoparticles (AgNPs) exhibited potent antimicrobial effects against clinically relevant Gram-negative pathogens, including *Escherichia coli*, *Klebsiella pneumoniae*, *Salmonella* Typhimurium, and *Salmonella* Enteritidis, demonstrating their potential as alternative antibacterial agents [13]. Presently, many nanoparticles have been approved by the American Food and Drug Administration (FDA) in chemotherapy, imaging agents, food supplements, etc. [14,15].

This study evaluated the antibacterial efficacy of various zinc–cobalt ferrite (Zn-CoFe_2_O_4_) nanoparticle formulations against a clinically relevant, multidrug-resistant *Escherichia coli* field strain. The selected strain exhibited a virulent phenotype characterized by virulence factors: *stx1* and *ehxA* genes;antibiotic resistance markers: *bla_AmpC_* and *bla_SHV_* (ESBL) genes; and biofilm production capacity mediated by *csgA*, *sdiA*, and *rpoS* genes [16].

## 2. Materials and Methods

### 2.1. Materials

**Precursors:** Zinc Nitrate [Zn (NO_3_)_2_·6H_2_O], Cobalt Nitrate [Co (NO_3_)_2_·6H_2_O], Ferric Nitrate [Fe (NO_3_)_3_·9H_2_O], Citric Acid (C_6_H_8_O_7_).

**Media:** All biological media used in this study were prepared as per manufacturers’ instructions and sterilized by autoclaving at 121 °C for 15 min under 15 psi pressures before use. To ensure sterility, all prepared bacteriological media and slants were incubated at 37 °C for 24 h prior to use.

**Bacterial Strains:** The bacterial field strain (isolated from duck) used in this study was obtained from the Department of Veterinary Microbiology, West Bengal University of Animal and Fishery Sciences (WBUAFS), Belgachia, Kolkata, India.

### 2.2. Methods

**Synthesis of Nanocrystalline [Zn_x_Co_(1−x)_Fe_2_O_4_]:** Nanocrystalline zinc–cobalt ferrite [Zn_x_Co_1−x_Fe_2_O_4_] nanoparticles were synthesized via a solution combustion method, as described by Sanpo et al. [7] with slight modifications. Metal nitrates of zinc [Zn(NO_3_)_2_·6H_2_O], cobalt [Co(NO_3_)_2_·6H_2_O], and iron [Fe(NO_3_)_3_·9H_2_O], along with citric acid (C_6_H_8_O_7_) as a fuel and chelating agent, were dissolved in distilled water to form a clear homogeneous solution. The solution was then heated to approximately 250 °C to facilitate gel formation. Upon gelation, the mixture was combusted at 850 °C for 2 h to yield the nanocrystalline zinc–cobalt ferrite powder.

Three different samples were synthesized by varying the Zn:Co molar ratio as follows: Sample 1(S1): Zn_0.7_Co_0.3_Fe_2_O_4_; Sample 2 (S2): Zn_0.5_Co_0.5_Fe_2_O_4_; Sample 3 (S3): Zn_0.3_Co_0.7_Fe_2_O_4_.

### 2.3. Physical Characteristics of Nanocrystalline Powder of Zinc–Cobalt Ferrite

**X-ray Diffraction:** The crystalline structure of the synthesized nanoparticles was characterized using X-ray diffraction (XRD) analysis. The measurements were carried out with a diffractometer equipped with Cu-Kα radiation (*λ* = 0.15406 nm), operating over a 2*θ* range of 10° to 90° [7].

The crystallite size (D) of the sample was estimated using the Debye–Scherrer formula,

$$D=\frac{0.9\lambda }{\beta \cos\theta }$$

where *λ* is the wavelength of X-ray radiation and *β* is the full width at half maximum of the peak, and *θ* is the Bragg angle.

The lattice parameter (*a*) was calculated according to the formula

$$a=d_{hkl}\sqrt{h^{2}+k^{2}+l^{2}}$$

where *d_hkl_* is the interplanar spacing and *h*, *k*, and *l* are Miller indices of the plane.

**Fourier-Transform Infrared Spectroscopy:** Fourier-transform infrared spectroscopy (FTIR) was employed to analyze the chemical bonds and confirm the spinel ferrite structure of the samples [17]. The ferrite powder was finely ground and mixed with potassium bromide (*KBr*) in a 1:100 ratio to form pellets. Spectra were recorded in the wavenumber range of 4500–500 cm^−1^.

**Scanning Electron Microscopy:** The surface morphology of the nanoparticles was examined using Scanning Electron Microscopy (SEM). Imaging was performed on a JEOL JSM-6380LA scanning electron microscope (Tokyo, Japan) operating at an accelerating voltage of 15 kV under high-vacuum conditions [18].

**Determination of Minimum Inhibitory Concentration and Minimum Bactericidal Concentration:** The antibacterial activity of the zinc–cobalt ferrite nanoparticles [Zn_x_Co_(1−x)_Fe_2_O_4_] was evaluated against Escherichia coli using the broth dilution method described by Azam et al. (2012) [12], with slight modifications. Briefly, the bacterial inoculums were adjusted to a concentration of 10^6^ CFU/mL by ten-fold serial dilution. For Minimum Inhibitory Concentration (MIC) determination, 10 mg of each synthesized nanoparticle sample was added to 1 mL of sterile distilled water and subjected to ultrasonication (5 cycles of 1 min, with 30 s intervals between cycles) to ensure homogenous dispersion. A 0.1 mL aliquot of the sonicated suspension was further diluted in 9.9 mL of sterile distilled water and sonicated again using the same protocol to obtain a working stock solution of 100 µg/mL. Serial two-fold dilutions were prepared in six sterile Eppendorf tubes (labeled 1–6), each containing 100 µL of normal saline solution (NSS). From the nanoparticle stock, 100 µL was added to tube 1 and serially diluted across the remaining tubes. Subsequently, 100 µL of the prepared *E. coli* suspension was added to each tube, mixed thoroughly, and incubated at 35 °C ± 2 °C for 24 h. The MIC was defined as the lowest concentration of the nanoparticle suspension that visibly inhibited bacterial growth.

For the determination of Minimum Bactericidal Concentration (MBC), aliquots from each MIC tube showing no visible growth were plated onto nutrient agar and incubated at 37 °C for 24 h. The MBC was recorded as the lowest concentration at which no bacterial colony formation was observed, indicating complete bactericidal activity.

**Well Diffusion and Disk Diffusion Assay:** The antibacterial activity of the synthesized zinc–cobalt ferrite nanoparticles was further evaluated using both the well diffusion and disk diffusion methods. The test solution was prepared by dispersing the nanoparticles in dimethyl sulfoxide (DMSO) to a final concentration of 1 mg/mL. To ensure homogeneity, the suspension was subjected to ultrasonication using a Hielscher ultrasonic processor (Teltow, Germany) equipped with a titanium probe, operating at 200 W with a duty cycle of 0.5 µs. The sonication was performed in 25 cycles of 1 min each, with 30 s intervals between cycles. *Escherichia coli* culture was evenly spread on Mueller–Hinton Agar (MHA) plates (Merck, Darmstadt, Germany) using sterile cotton swabs to create a uniform lawn of bacterial growth. Gentamicin and amoxicillin disks were used as the positive and negative control, respectively. For the well diffusion assay, 8 mm wells were aseptically punched into the agar, and each well was filled with either 50 µL or 100 µL of the nanoparticle test solution. For the disk diffusion assay, two sterile blank disks were impregnated with 50 µL and 100 µL of test solution and placed on the inoculated agar surface. All plates were incubated at 37 °C for 24 h. Post incubation, zones of inhibition (in mm) around the wells and disks were measured to assess the antibacterial efficacy of the nanoparticles.

## 3. Results

### 3.1. Physical Characteristics of Nanocrystalline Powder of Zinc Cobalt Ferrite

#### 3.1.1. Scanning Electron Micrograph Analysis

**Figure 1:**
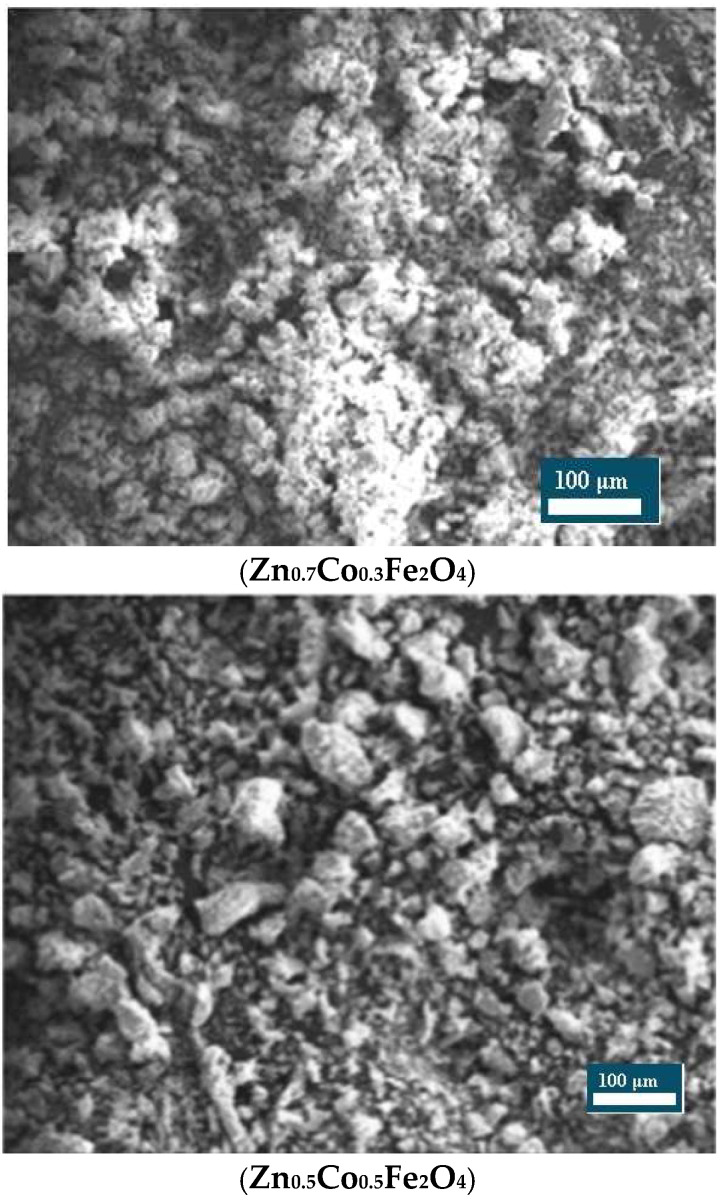

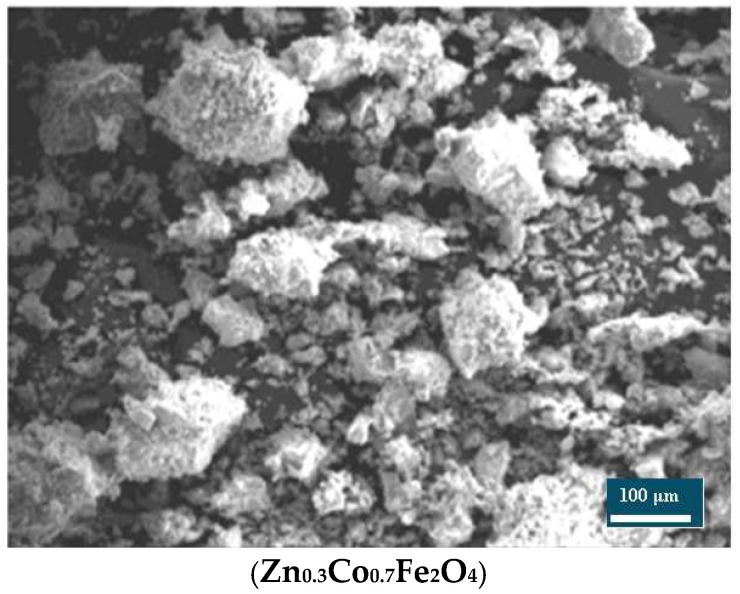
SEM analysis of zinc–cobalt ferrite nanoparticles.

The surface morphology of zinc–cobalt ferrite nanoparticles, as shown in Figure 1, demonstrated distinct variations correlated with cobalt substitution levels within the spinel ferrite matrix. Scanning Electron Micrographs (SEMs) of Zn_0.7_Co_0.3_Fe_2_O_4_ revealed relatively smooth surfaces with moderately agglomerated, nearly spherical nanoparticles. With an increase in cobalt content to Zn_0.5_Co_0.5_Fe_2_O_4_, the particles exhibited denser packing, along with increased agglomeration and irregularity in shape. At the highest cobalt substitution level, Zn_0.3_Co_0.7_Fe_2_O_4_, the microstructure appeared significantly more irregular, characterized by pronounced agglomeration and a wider particle size distribution.

#### 3.1.2. X-Ray Diffraction Analysis

**Figure 2:**
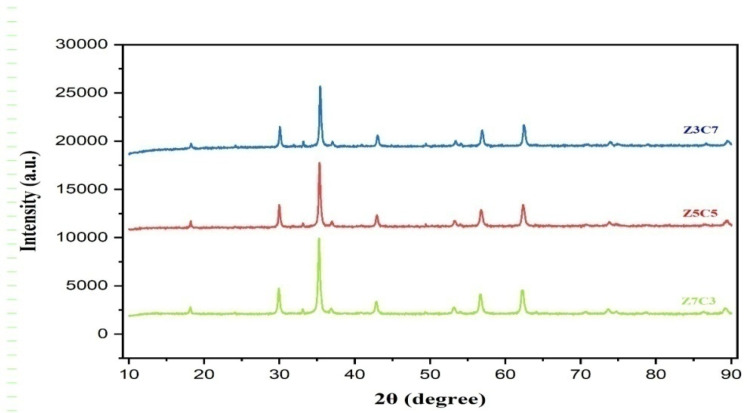
XRD analysis of zinc–cobalt ferrite nanoparticles.

The X-ray diffraction (XRD) patterns of the synthesized Zn_x_Co_(1−x)_Fe_2_O_4_ (where *x* = 0.7, 0.5, 0.3) nanoparticles recorded in the 2θ range of 10° to 90° are presented in Figure 2. The distinct diffraction peaks confirmed the formation of a pure single-phase spinel Zn-Co Ferrite with a face-centered cubic (FCC) structure. No secondary phases or impurity peaks were detected, indicating high phase purity. The characteristic diffraction peaks were observed at 2θ values of approximately 30.1°, 35.5°, 43.1°, 53.4°, 57.0°, and 62.6°, corresponding to the (220), (311), (400), (422), (511), and (440) planes, respectively. Among these, the (311) plane exhibited the highest intensity and was employed to calculate both crystallite size and lattice constant.

The calculated crystallite sizes and lattice constants are summarized in Table 1. A trend of increasing crystallite size was observed with higher cobalt substitution, ranging from 53.236 nm (S1, i.e., Zn_0.7_Co_0.3_Fe_2_O_4_) to 53.987 nm (S3, i.e., Zn_0.3_Co_0.7_Fe_2_O_4_). This increase is attributed to enhanced grain growth facilitated by the higher Co^2+^ content, which affects nucleation rates and growth kinetics during the combustion synthesis process. Conversely, the lattice constant was found to decrease with increasing cobalt content, from 84.3 nm to 83.9 nm, consistent with Vegard’s law. This decrease is due to the replacement of larger Zn^2+^ ions (0.60 nm) by smaller Co^2+^ ions (0.58 nm), leading to slight lattice contraction.

#### 3.1.3. Fourier-Transform Infrared Analysis

**Figure 3:**
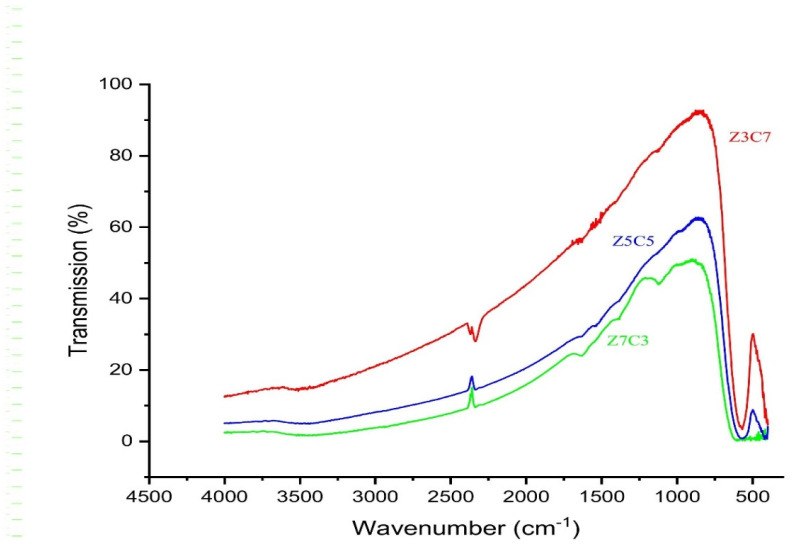
FTIR analysis of zinc–cobalt ferrite nanoparticles.

The Fourier-transform infrared (FTIR) spectra of the synthesized Zn_x_Co_(1−x)_Fe_2_O_4_ nanoparticles is shown in Figure 3. Characteristic absorption bands were observed in the range of 400–600 cm^1^, corresponding to the stretching vibration of metal–oxygen bonds (ZnO, CoO, Fe_2_O_3_). These peaks confirm the successful formation of the cubic spinel ferrite structure. In particular, the higher-frequency band (typically near 570–590 cm^−1^) is attributed to metal–oxygen stretching vibrations at tetrahedral sites, while the lower-frequency band (typically near 430–480 cm^−1^) corresponds to stretching vibrations at octahedral sites within the spinel lattice. These two distinct bands are indicative of the spinel structure, where the distribution of metal ions between tetrahedral and octahedral sites plays a critical role in determining the material’s physical and chemical properties. The FTIR data thus provides further confirmation of the successful synthesis of nanocrystalline Zn_x_Co_(1−x)_Fe_2_O_4_ with a spinel ferrite structure.

**Antibacterial Activity:** This study aimed to evaluate the antibacterial efficacy of various formulations of zinc–cobalt ferrite (Zn_x_Co_(1−x)_Fe_2_O_4_) nanoparticles against *Escherichia coli*. The Minimum Inhibitory Concentration (MIC) and Minimum Bactericidal Concentration (MBC) values for each nanoparticle sample were determined based on turbidity in tubes and specific bacterial growth on culture plates, respectively. The lowest concentrations of the samples giving no turbidity in Eppendorf tubes and no growth on culture plates was considered for the MIC and MBC values. The MIC and MBC values were found to be: Zn_0.7_Co_0.3_Fe_2_O_4_: 12.5 µg/mL, Zn_0.5_Co_0.5_Fe_2_O_4_: 12.5 µg/mL, and Zn_0.3_Co_0.7_Fe_2_O_4_: 50 µg/mL. These findings clearly demonstrate the potent antibacterial activity of Zn-Co ferrite (Zn_x_Co_(1−x)_Fe_2_O_4_) nanoparticles, particularly for the samples with a higher zinc content. The lower MIC and MBC values for Zn_0.7_Co_0.3_Fe_2_O_4_ and Zn_0.5_Co_0.5_Fe_2_O_4_ suggest greater efficacy in inhibiting and eliminating *E. coli*, likely due to enhanced physicochemical interactions between the nanoparticle surface and bacterial cell membranes. In contrast, the reduced activity observed in Zn_0.3_Co_0.7_Fe_2_O_4_ may be attributed to increased agglomeration and surface irregularities at higher cobalt concentrations, as revealed by SEM analysis. A comparative summary of the MIC and MBC values is provided in Table 2.

## 4. Discussion

The calculated crystallite sizes of S1 (Zn_0.7_Co_0.3_Fe_2_O_4_), S2 (Zn_0.5_Co_0.5_Fe_2_O_4_), and S3 (Zn_0.3_Co_0.7_Fe_2_O_4_) were 21.65 nm, 24.53 nm, and 25.39 nm, respectively. The present findings are in agreement with the observations of Vinuthna et al. [17], who reported the crystallite size of zinc–cobalt ferrite nanoparticles in the range of 22–29 nm. In contrast, Abdel Maksoud et al. [18], documented a smaller crystallite size of approximately 12.86 nm for similar materials. Such discrepancies in crystallite size may be attributed to differences in synthesis methodologies, particularly in factors like combustion temperature, precursor ratios, and duration of calcination, all of which significantly influence particle growth and structural properties.

The Fourier-transform infrared (FTIR) spectra of the synthesized Zn_x_Co_(1−x)_Fe_2_O_4_ nanoparticles show the characteristic absorption bands only for metal–oxygen stretching vibration in tetrahedral and octahedral sites which confirm the formation of the cubic spinel ferrite structure. There is a very slight shifting of peaks with the increase in Zn^+2^ content in the host lattice but it is not too remarkable a change. This slight change is due to the presence of larger size Zn^+2^ in tetrahedral sites and Fe^+3^ in octahedral sites [19].

The MIC and MBC values of Zn_0.7_Co_0.3_Fe_2_O_4_, Zn_0.5_Co_0.5_Fe_2_O_4_, and Zn_0.3_Co_0.7_Fe_2_O_4_ were found to be 12.5 µg/mL, 12.5 µg/mL, and 50 µg/mL, respectively. The lowest concentrations of the samples giving no turbidity in the Eppendorf tubes and no growth on the culture plates were considered for the MIC and MBC values. These findings clearly demonstrate the potent antibacterial activity of Zn-Co Ferrite (Zn_x_Co_(1−x)_Fe_2_O_4_) nanoparticles, particularly for samples with a higher zinc content. The present findings corroborate earlier reports demonstrating significant antibacterial efficacy of zinc ferrite nanopowders against *E. coli* [11], superparamagnetic ZnFe_2_O_4_ nanoparticles [20], and zinc–cobalt ferrite against multiple bacterial strains including *E. coli* [18]. The enhanced antibacterial activity observed, particularly in samples with a higher zinc content, may be attributed to several proposed mechanisms associated with metal ferrite nanoparticles. One of the primary mechanisms involves the generation of reactive oxygen species (ROS) such as hydroxyl radicals (OH), superoxide anions (O_2_^−^), and hydrogen peroxide (H_2_O_2_), which can cause oxidative damage to bacterial cell membranes, proteins, and nucleic acids. The nanoparticles may catalyze the Fenton-like reaction, leading to ROS generation, thereby inducing membrane lipid peroxidation and disrupting membrane integrity.

Another plausible mechanism is the direct interaction of nanoparticles with the bacterial cell envelope. Zinc and cobalt ions released from the nanoparticles can bind to negatively charged components of the bacterial membrane, leading to increased membrane permeability, ion imbalance, and eventual cell lysis. The nanoscale size also facilitates close interaction with the bacterial surface, enhancing the material’s bactericidal efficiency. Furthermore, Zn^2+^ ions are known to interfere with essential enzymatic functions and metabolic pathways inside the bacterial cells, while Co^2+^ ions may contribute to electron transfer imbalances, disrupting cellular respiration. The substitution of *Zn* and *Co* in the ferrite matrix may also modulate surface charge and particle stability, thereby influencing nanoparticle–bacteria interactions. Thus, the combined effects of physical disruption, ROS-mediated oxidative stress, and ion-induced metabolic interference underlie the antibacterial activity of Zn_x_Co_(1–x)_Fe_2_O_4_ nanoparticles. The variation in MIC and MBC values among the different compositions is likely due to the differences in their surface morphology, particle size, and elemental composition, which collectively influence their reactivity and interaction with bacterial cells.

In the present study, however, clear zones of inhibition were not observed in either the well diffusion or disk diffusion assays for *E. coli*, despite the demonstrated antibacterial activity in MIC and MBC tests. This apparent discrepancy can be primarily attributed to the poor aqueous solubility and rapid precipitation of the zinc cobalt ferrite nanoparticles. Upon placement in the wells or on disks, the nanoparticles tended to aggregate and settle, limiting their diffusion through the agar medium. As a result, the active antibacterial agents could not effectively migrate to the surrounding bacterial lawn, thereby failing to inhibit bacterial growth in a spatial manner. This limitation is consistent with the known challenge of poor dispersibility and mobility of metal oxide nanoparticles in agar-based assays, which often leads to underestimation of their true antimicrobial potential. Therefore, MIC and MBC assays, which allow for better suspension and contact between nanoparticles and bacterial cells, may provide a more accurate assessment of their antibacterial efficacy.

The precise mechanisms by which metal ferrite nanoparticles exert bactericidal effects remain to be fully elucidated. However, several studies propose that their nanoscale dimensions facilitate cellular penetration and subsequent induction of oxidative stress through free radical and reactive oxygen species (ROS) generation. Specifically, released Zn^2+^ ions can competitively inhibit Mn^2+^ uptake—an essential cofactor for numerous bacterial enzymes—thereby disrupting metabolic processes and leading to cell death. Concurrently, Co^2+^ ions have been shown to intercalate with bacterial DNA, causing strand breaks and impairing replication. Additional factors influencing the antibacterial efficacy of nanocrystalline Zn–Co ferrite include nanoparticle concentration, bacterial species, morphology, and cell wall architecture [13]. Mechanistic models such as the “Barrel-Stave” and “Carpet” membrane disruption pathways have been postulated, wherein nanoparticles initially associate with the negatively charged outer membrane and subsequently form transmembrane channels or destabilize lipid bilayers, culminating in cytoplasmic leakage and cell lysis [11].

## 5. Conclusions

In this study, nanocrystalline zinc–cobalt ferrite (Zn_x_Co_(1−x)_Fe_2_O_4_) nanoparticles were successfully synthesized using the solution combustion technique and systematically characterized for their structural, morphological, and antibacterial properties. Among the different compositions tested, Zn_0.7_Co_0.3_Fe_2_O_4_ exhibited the highest antibacterial efficacy against virulent, multidrug-resistant, and biofilm-forming *Escherichia coli*. This enhanced activity is attributed to the optimized surface morphology, crystallite size, and chemical composition that favor interactions with bacterial membranes. The study further suggests that modifying the Zn:Co molar ratio plays a critical role in tuning the antibacterial properties of the material. While traditional zone of inhibition methods showed limited results—likely due to the poor solubility and mobility of the particles in agar medium—the MIC and MBC assays demonstrated effective bacterial growth inhibition and killing at relatively low concentrations, particularly in samples with a higher zinc content.

These findings underscore the potential of Zn-Co ferrite nanoparticles as promising candidates for the development of next-generation antimicrobial agents. Their ability to disrupt bacterial physiology, possibly through reactive oxygen species (ROS) generation, ion release, and membrane interaction, offers an alternative strategy to combat the growing threat of antibiotic-resistant pathogens. Further investigations, including in vivo studies and biocompatibility assessments, are warranted to fully explore their clinical applicability.

## Figures and Tables

**Table 1:** Data on crystallite size (D) and lattice constant of Zn-Co ferrite nanoparticles.

Sample	Crystallite Size (nm)	Lattice Constant (nm)
S1 (Zn_0.7_Co_0.3_Fe_2_O_4_)	53.276	84.3
S2 (Zn_0.5_Co_0.5_Fe_2_O_4_)	53.782	84.2
S3 (Zn_0.3_Co_0.7_Fe_2_O_4_)	53.987	83.9

**Table 2:** Determination of MIC and MBC of different samples of test material.

Samples	Concentration(µg/mL)	Observation (Turbidity in Tube)	MIC (µg/mL)	Observation (Bacterial Growth in Plate)	MBC (µg/mL)
Zn_0.7_Co_0.3_Fe_2_O_4_	50	No Turbidity	12.5	No Growth	12.5
	25	No Turbidity		No Growth	
	12.5	No Turbidity		No Growth	
	6.25	Turbidity		Growth	
	3.12	Turbidity		Growth	
	1.56	Turbidity		Growth	
Zn_0.5_Co_0.5_Fe_2_O_4_	50	No Turbidity	12.5	No Growth	12.5
	25	No Turbidity		No Growth	
	12.5	No Turbidity		No Growth	
	6.25	Turbidity		Growth	
	3.12	Turbidity		Growth	
	1.56	Turbidity		Growth	
Zn_0.3_Co_0.7_Fe_2_O_4_	50	No Turbidity	50	No Growth	50
	25	Turbidity		Growth	
	12.5	Turbidity		Growth	
	6.25	Turbidity		Growth	
	3.12	Turbidity		Growth	
	1.56	Turbidity		Growth	

## Data Availability

All data generated or analyzed during this study are included in this published article.
